# Comparable Bioavailability and Disposition of Pefloxacin in Patients with Cystic Fibrosis and Healthy Volunteers Assessed via Population Pharmacokinetics

**DOI:** 10.3390/pharmaceutics11070323

**Published:** 2019-07-10

**Authors:** Jürgen B. Bulitta, Yuanyuan Jiao, Cornelia B. Landersdorfer, Dhruvitkumar S. Sutaria, Xun Tao, Eunjeong Shin, Rainer Höhl, Ulrike Holzgrabe, Ulrich Stephan, Fritz Sörgel

**Affiliations:** 1 Department of Pharmacotherapy and Translational Research, College of Pharmacy, University of Florida, Orlando, FL 32827, USA; 2 Drug Delivery, Disposition and Dynamics, Monash Institute of Pharmaceutical Sciences, Monash University, Parkville VIC 3052, Australia; 3 Institute of Clinical Hygiene, Medical Microbiology and Infectiology, Klinikum Nürnberg, Paracelsus Medical University, 90419 Nürnberg, Germany; 4 Institute for Pharmacy and Food Chemistry, University of Würzburg, 97074 Würzburg, Germany; 5 IBMP—Institute for Biomedical and Pharmaceutical Research, 90562 Nürnberg-Heroldsberg, Germany; 6 Department of Pharmacology, University of Duisburg, 47057 Essen, Germany

**Keywords:** cystic fibrosis patients, healthy volunteers, fluoroquinolone, pefloxacin, absolute bioavailability, population pharmacokinetics, allometric scaling, body size, body composition, S-ADAPT

## Abstract

Quinolone antibiotics present an attractive oral treatment option in patients with cystic fibrosis (CF). Prior studies have reported comparable clearances and volumes of distribution in patients with CF and healthy volunteers for primarily renally cleared quinolones. We aimed to provide the first pharmacokinetic comparison for pefloxacin as a predominantly nonrenally cleared quinolone and its two metabolites between both subject groups. Eight patients with CF (fat-free mass [FFM]: 36.3 ± 6.9 kg, average ± SD) and ten healthy volunteers (FFM: 51.7 ± 9.9 kg) received 400 mg pefloxacin as a 30 min intravenous infusion and orally in a randomized, two-way crossover study. All plasma and urine data were simultaneously modelled. Bioavailability was complete in both subject groups. Pefloxacin excretion into urine was approximately 74% higher in patients with CF compared to that in healthy volunteers, whereas the urinary excretion of metabolites was only slightly higher in patients with CF. After accounting for body size and composition via allometric scaling by FFM, pharmacokinetic parameter estimates in patients with CF divided by those in healthy volunteers were 0.912 for total clearance, 0.861 for nonrenal clearance, 1.53 for renal clearance, and 0.916 for volume of distribution. Nonrenal clearance accounted for approximately 90% of total pefloxacin clearance. Overall, bioavailability and disposition were comparable between both subject groups.

## 1. Introduction

The pharmacokinetics (PK) of patients with cystic fibrosis (CF) has been recently compared to that in healthy volunteers for multiple classes of antibiotics [1,2,3]. Quinolone antibiotics present an attractive oral treatment option for patients with CF. Several studies compared the PK of quinolones between patients with CF and healthy volunteers for ciprofloxacin [4,5,6,7,8,9], fleroxacin [10,11] and levofloxacin [12]. These quinolones are relatively hydrophilic with distribution coefficients (log D) of −1.24 for ciprofloxacin, −0.76 for fleroxacin and −0.50 for levofloxacin; and they are primarily renally eliminated [13,14,15,16]. Most of these studies found comparable total and renal clearances and similar or slightly smaller nonrenal clearances in patients with CF compared to those in healthy volunteers [4,6,9,10,11,12]. One study with a small sample size reported a larger renal clearance of ciprofloxacin [5]. Moreover, the renal clearances of two fleroxacin metabolites (i.e., *N*-oxide fleroxacin and *N*-demethylfleroxacin) were 53% and 70% larger based on a population PK analysis which accounted for body size and composition [11]. The mechanism for the higher renal clearance of these fleroxacin metabolites is not known.

Pefloxacin is a more lipophilic quinolone with a log D of 0.20 and is predominantly nonrenally cleared. We are not aware of prior studies which compared the PK of a primarily nonrenally cleared quinolone between patients with CF and healthy volunteers. The bioavailability of pefloxacin approaches 100% and its protein binding is approximately 25% in human plasma [13,17,18,19]. Most studies report a total clearance between 5.2 and 8.3 L/h, terminal half-life between 9.7 and 13.7 h, and volume of distribution between approximately 100 and 140 L; nonrenal clearance is the predominant elimination pathway. Pefloxacin has been studied in-depth in the 1980s and 1990s [20,21], but has not been approved in the United States and was withdrawn from the market in other countries. Some studies showed that pefloxacin caused a higher risk of tendon damage compared to that of other quinolones [22,23,24]; economic reasons likely further contributed to pefloxacin falling out of favor. However, pefloxacin presents a suitable probe drug to compare the PK and nonrenal clearance between patients with CF and healthy volunteers.

Two studies [7,8] on ciprofloxacin compared the PK in pediatric patients with and without CF via population PK modeling; one of these studies [7] described body size by an allometric model based on total body weight (WT). In adult patients with CF, all but one study on quinolones [11] employed non-compartmental PK analysis to compare the PK in patients with CF to that in healthy volunteers [2,3]. Population PK modeling offers the advantage that it can simultaneously describe the population mean PK parameters and their between subject variability (BSV) for the parent drug and metabolites. Moreover, the effect of body size and body composition can be incorporated via allometric scaling [25] based on WT or fat-free mass (FFM) [26]. The ability to account for body size and estimate the effect of CF presents a considerable advantage of population PK modeling [27,28,29,30,31].

The present study aimed to compare the bioavailability and disposition between adult patients with CF and healthy volunteers for pefloxacin and its two metabolites, norfloxacin and pefloxacin *N*-oxide, via population PK modeling. Pefloxacin was dosed intravenously and orally in a randomized, two-way crossover study. Bioavailability was complete in both subject groups. We used an allometric body size model based on FFM to account for the differences in body size and body composition between patients with CF and healthy volunteers. The body size adjusted total clearance, nonrenal clearance and volume of distribution at steady-state were well comparable between both subject groups (i.e., 8%–14% smaller in patients with CF). However, renal clearance of pefloxacin was considerably (53%) larger in patients with CF. This presents the first study that compared the PK of a primarily nonrenally cleared quinolone between patients with CF and healthy volunteers.

## 2. Methods and Materials

*Subjects*: A total of 18 Caucasian volunteers (eight patients with CF and ten healthy volunteers) participated in the study (Table 1). The health status of subjects was assessed by electrocardiography, physical examination, and laboratory tests including urinalysis and screening for drugs of abuse. The consumption of alcohol and methylxanthines in any form was forbidden from 12 h before each pefloxacin dose until the last sample. The subjects fasted overnight and received a standardized breakfast at 1 h, lunch at 4 h, and dinner at 12 h post dose. Sufficient fluid intake of mineral water was assured. The study protocol had been approved by the ethics committee of the University Hospital Essen under the title “Pharmakokinetik von Antibiotika bei Mukoviszidose-Patienten und gesunden Probanden” (approved on 29 August 1984). The study was conducted according to the revised version of the Declaration of Helsinki. All volunteers had given their written informed consent before they were enrolled in the study. One patient with CF was 17 years old and written informed consent was obtained from his legal representative.

*Study design*: The study was a randomized, single dose, single-center, open, two-way crossover with a washout period of 10 days. Subjects received 400 mg pefloxacin as 30 min intravenous infusion and 400 mg oral pefloxacin (i.e., a standard dose of pefloxacin) in either study period. For oral dosing, subjects took pefloxacin with 150 mL of low-carbonated, calcium-poor mineral water at room temperature. Patients with CF abstained from taking pancreatic enzymes as supplement therapy from at least 10 h before until 4 h after the pefloxacin dose. For the intravenous infusion, 400 mg pefloxacin were dissolved in 300 mL of glucose solution (5%) and this mixture was shaken thoroughly for 2–3 min. All infusions were administered with exactly adjustable motor syringes which were checked on a daily basis by weighing defined volumes delivered by the motor syringes.

*Blood sampling*: All blood samples were drawn from a forearm vein via an intravenous catheter which was placed contralateral to the one used for drug dosing (in case of intravenous treatment). For intravenous dosing, the blood samples were drawn immediately before the beginning of the infusion and at 10, 20, and 30 min post start of infusion; additional blood samples were drawn at 5, 10, 20, 30, 45, 60, 90 min as well as 2, 2.5, 3, 4, 5, 6, 8, 10, 12, 16, 24, 30, 36, and 48 h after the end of the infusion. For oral administration, blood samples were drawn immediately before dosing and at 15, 30, 45, 60, 90 min as well as 2, 3, 4, 6, 8, 10, 12, 16, 24, 30, 36, and 48 h after administration. The samples were immediately centrifuged, frozen and stored at −20 °C until analysis.

*Urine collections*: Urine samples were collected from 0 to 1, 1 to 2, 2 to 3, 3 to 4, 4 to 6, 6 to 8, 8 to 12, 12 to 16, 16 to 24, 24 to 36, and 36 to 48 h after dosing. At the end of the collection intervals the amount of urine was measured and aliquots were immediately frozen and stored at −20 °C until analysis.

*Drug analysis*: Plasma samples were analyzed for pefloxacin. In urine samples, we quantified pefloxacin and its main metabolites, pefloxacin *N*-oxide and norfloxacin, via reversed-phase high-performance liquid chromatography (HPLC) assays which have been described previously [32]. The mobile phase consisted of 50% methanol and 50% 0.1 M phosphate buffer (at pH 4.9) for determining pefloxacin in plasma which had a retention time of 5.0 min. The reversed-phase column (Nucleosil C18 5-µm) was heated to 40 °C (Bischoff GmbH, Leonberg, Germany) and used as stationary phase. Plasma samples were deproteinized by addition of acetonitrile (1:2). The resulting supernatant was injected into the mobile phase at a flow rate of 1.2 mL/min. The mobile phase consisted of 24.1% methanol, 2.6% acetonitrile, and 73.3% 0.1 M phosphate buffer (pH 5.75) for pefloxacin concentrations and those of its metabolites in urine. In the latter matrix, a C18 µ-Bondapak reversed-phase column (Waters Association, Eschborn, Germany) was employed. The urine samples were diluted with double-distilled water before injection into the mobile phase using a flow rate of 1.0 mL/min. For urine samples, the retention time was approximately 3 min for pefloxacin, 5 min for norfloxacin and 10 min for pefloxacin *N*-oxide.

For quantification of pefloxacin, pefloxacin *N*-oxide, and norfloxacin, fluorescence was measured by a Perkin Elmer 650-10 LC Fluorescence Spectrometer (Perkin-Elmer, Überlingen, Germany). The excitation wavelength was 275 nm with emission at 415 nm. The linear range of the assay ranged from 0.078 to 20 mg/L for pefloxacin in plasma, from 0.78 to 100 mg/L for pefloxacin in urine, and from 3.13 to 200 mg/L for the metabolites in urine. The coefficients of correlation exceeded 0.999. The within-day precision of the assay was 3.2% at 2.5 mg/L and 6.9% at 0.63 mg/L pefloxacin in plasma (coefficients of variation [CV]). These CVs were 3.1% at 100 mg/L and 5.1% at 12.5 mg/L pefloxacin in urine. The between-day precision had CVs of 2.7% at 3.8 mg/L and 3.4% at 1.5 mg/L for pefloxacin in plasma, as well as 3.8% at 40.2 mg/L and 7.3% at 8.3 mg/L for pefloxacin in urine.

### Population Pharmacokinetic Analysis

**Figure 1:**
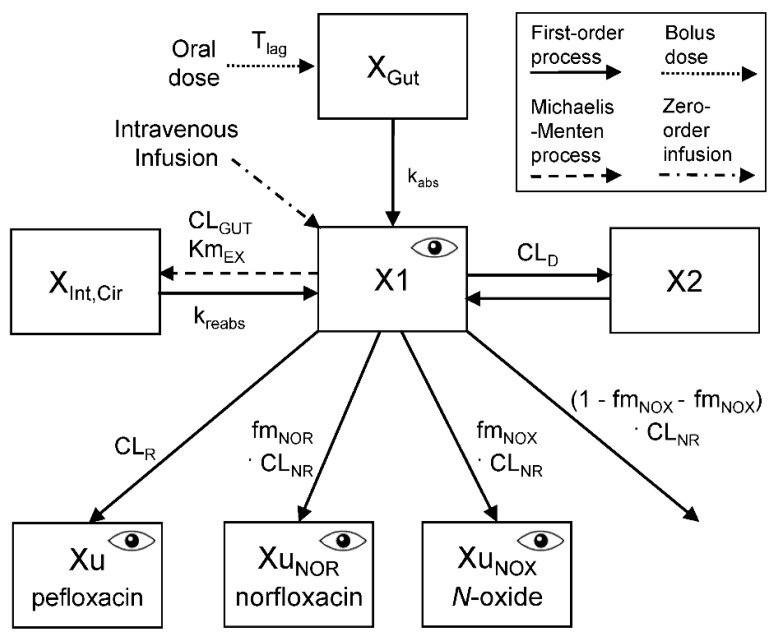
Structural model for pefloxacin, norfloxacin and pefloxacin *N*-oxide in plasma and urine.

*Structural model*: One, two, and three compartment disposition models were tested. Oral absorption was described as a first-order process (rate constant: k_abs_) from the gut into the central compartment (with or without a lag-time). The intravenous infusion was described by a time-delimited zero-order input rate (R_Inf_) into the central compartment. The extent of oral bioavailability (F) and its BSV were estimated based on data after oral and intravenous dosing in each subject. Renal (CL_R_) and nonrenal clearance (CL_NR_) of pefloxacin were included. Additionally, the final model (Figure 1) contained an intestinal recirculation compartment with a saturable exsorption clearance (CL_EX_) and subsequent reabsorption (rate constant: k_reabs_). The differential equations were:

(1) $$\frac{{\mathrm{dX}}_{\mathrm{Gut}}}{\mathrm{dt}}=-{\;k}_{\mathrm{abs}}\cdot X_{\mathrm{Gut}}$$

(2) $$\frac{\mathrm{dX}1}{\mathrm{dt}}=R_{\mathrm{Inf}}+{\;k}_{\mathrm{abs}}\cdot X_{\mathrm{Gut}}-\left({\mathrm{CL}}_{R}+{\mathrm{CL}}_{\mathrm{NR}}+{\mathrm{CL}}_{\mathrm{EX}}\right)\cdot C1-{\;\mathrm{CL}}_{D}\;\cdot \left(C1-C2\right)+k_{\mathrm{reabs}}\cdot X_{\mathrm{Int},\mathrm{Cir}}$$

(3) $$\frac{\mathrm{dX}2}{\mathrm{dt}}={\mathrm{CL}}_{D}\;\cdot \left(C1-C2\right)$$

where X_Gut_ is the amount of pefloxacin in the gut compartment, X1 the amount of pefloxacin in the central and X2 the amount in the peripheral compartment. The C1 and C2 are the pefloxacin concentrations in the central and peripheral compartments and CL_D_ is the distribution clearance. 

*Intestinal exsorption and reabsorption*: We explored a potential exsorption of pefloxacin from the central compartment into intestine which has been previously reported for fleroxacin and other quinolones using charcoal studies [11,13,14,33]. For some quinolones, the amount of parent drug recovered in feces [33,34] is much larger than the fraction of dose recovered in bile [13,14]. This suggests the presence of exsorption from the central circulation into intestine.

An additional intestine compartment was included to describe the amount of pefloxacin (X_Int,Cir_) that was exsorbed into intestine and subsequently reabsorbed into the central compartment. Both a linear and saturable exsorption clearance (CL_EX_) from the central into the intestinal recirculation compartment were explored. The saturable exsorption clearance (CL_EX_) was described by a maximum exsorption clearance and a Michaelis-Menten constant (Km_EX_) which is the concentration in the central compartment associated with a half-maximal exsorption rate. The maximum excretion clearance was fixed to the blood flow to gut (CL_GUT_, 66 L/h) for subjects of normal body size [35]; models which estimated blood flow to the gut were additionally explored. The product of CL_GUT_ (unit: L/h) and Km_EX_ (unit: mg/L) equals the maximum rate of exsorption (Vmax_EX_; unit: mg/h).

(4) $${\mathrm{CL}}_{\mathrm{EX}}=\;\frac{{\mathrm{Vmax}}_{\mathrm{EX}}\;}{{\mathrm{Km}}_{\mathrm{EX}}+C1}$$

(5) $$\frac{{\mathrm{dX}}_{\mathrm{Int},\mathrm{Cir}}}{\mathrm{dt}}={\;\mathrm{CL}}_{\mathrm{EX}}\cdot C1-{\;k}_{\mathrm{reabs}}\cdot X_{\mathrm{Int},\mathrm{Cir}}$$

Given the complete bioavailability of pefloxacin, all of the exsorbed pefloxacin was modeled to be reabsorbed. Separate compartments were used for X_Gut_ and X_Int,Cir_, to allow for different absorption rate constants during oral dosing of pefloxacin in the morning and during reabsorption throughout the day. Moreover, the k_reabs_ was allowed to differ between patients with CF and healthy volunteers.

*Metabolites formation*: The amounts of the two metabolites, norfloxacin and pefloxacin *N*-oxide, in urine were used to estimate the fractions of the nonrenal clearance of pefloxacin that led to formation of norfloxacin (fm_NOR_) and pefloxacin *N*-oxide (fm_NOX_; Figure 1). Plasma concentrations of the metabolites were not available and therefore the clearance of the metabolites could not be estimated. We assumed that the metabolites were predominantly renally eliminated to calculate the metabolite formation clearances based on urinary excretion data of the metabolites. The estimation code for the three components of nonrenal clearance of pefloxacin (Figure S3) assured that the sum of all three fractions was 100% based on a logistic transformation.

*Urinary excretion*: Three compartments were included for the amounts of pefloxacin, norfloxacin and pefloxacin *N*-oxide in urine. The fractions of pefloxacin and its metabolites excreted into urine were modelled based on the cumulative amount of the respective compound excreted until the last collection interval, since the individual amounts of pefloxacin and its metabolites in each urine collection interval were not available. Modeling the total urinary excretion data for the three compounds in each subject was sufficient to estimate fm_NOR_ and fm_NOX_. The formation clearance for each metabolite was calculated as the product of the fm_NOR_ and fm_NOX_ with the nonrenal clearance of pefloxacin.

(6) $$\frac{{\mathrm{dX}}_{U}}{\mathrm{dt}}={\mathrm{CL}}_{R}\cdot C1$$

(7) $$\frac{{\mathrm{dX}}_{U,\;\mathrm{NOR}}}{\mathrm{dt}}={\mathrm{fm}}_{\mathrm{NOR}}\cdot {\mathrm{CL}}_{\mathrm{NR}}\cdot C1$$

(8) $$\frac{{\mathrm{dX}}_{U,\;\mathrm{NOX}}}{\mathrm{dt}}={\mathrm{fm}}_{\mathrm{NOX}}\cdot {\mathrm{CL}}_{\mathrm{NR}}\cdot C1$$

*Body size and composition*: We compared five models to describe body size and body composition: (1) No size model, (2) linear scaling by WT, (3) allometric scaling by WT [25], (4) linear scaling by FFM, and (5) allometric scaling by FFM [26]. The ability of each body size model to describe the differences in the central tendency of PK parameters between patients with CF and healthy volunteers was evaluated. An ideal body size model should explain the differences in the average PK parameter estimates between patients with CF and healthy volunteers. Moreover, we studied how much of the random BSV was explained (i.e., reduced) by the respective body size model.

The allometric body size models assume that volume of distribution scales linearly (exponent 1.0) with body size (i.e., WT or FFM) and that clearance scales less than linearly (allometric exponent 0.75) with body size. We fixed the allometric exponent to 1.0 for all volumes and to 0.75 for all clearances. The F_Size,V,i_ and F_Size,CL,i_ represent the relative changes in volume of distribution and in clearance of the i^th^ subject (WT_i_) standardized to a standard body weight (WT_STD_ of 70 kg). The same allometric size model was used for FFM with a standard fat-free mass FFM_STD_ of 53 kg. For linear scaling by WT and FFM (size model 2 and 4) all exponents were set to 1.0.

(9) $$F_{\mathrm{Size},V,i}=\frac{{\mathrm{FFM}}_{i}}{{\mathrm{FFM}}_{\mathrm{STD}}}$$

(10) $$F_{\mathrm{Size},\mathrm{CL},i}={\left(\frac{{\mathrm{FFM}}_{i}}{{\mathrm{FFM}}_{\mathrm{STD}}}\right)}^{0.75}$$

*Between-subject variability model*: We estimated BSV of PK parameters by log-normal distributions and reported the apparent coefficients of variation (i.e., the square roots of the estimated variances). The individual renal clearance (CL_R,i_) in the i^th^ subject, for example, was calculated as:

(11) $${\mathrm{CL}}_{R,\;i}={\;\mathrm{CL}}_{\mathrm{POP},\;R}\cdot F_{\mathrm{Size},\;\mathrm{CL},\;i}\cdot F_{\mathrm{CYF},\;\mathrm{CLR}}\cdot \exp\left(\eta_{\mathrm{BSV},\;\mathrm{CLRi}}\right)$$

The CL_POP,R_ is the population mean for renal clearance in healthy volunteers with a standard body size (i.e., WT_STD_ = 70 kg or FFM_STD_ = 53 kg). The η_BSV,CLRi_ is the random deviate of CL_R_ for the i^th^ subject. The F_CYF, CLR_ is the disease specific scale factor for renal clearance and represents the size-adjusted renal clearance in patients with CF divided by that in healthy volunteers. An F_CYF, CLR_ of 1.0 indicates, after accounting for body size and body composition, that patients with CF and healthy volunteers have identical group estimates for renal clearance. Similar scale factors were estimated for nonrenal clearance (F_CYF, CLNR_) and the volume of distribution at steady-state (F_CYF, VSS_). The scale factor for total clearance was calculated as a weighted average between F_CYF, CLR_ and F_CYF, CLNR_. 

*Residual error model and uncertainty*: We described the residual unidentified variability by a combined additive plus proportional residual error model for plasma concentrations of pefloxacin. We only had data available for the cumulative excretion of pefloxacin and its metabolites into urine in each study period. Therefore, the additive residual errors for the fractions of dose excreted into urine were fixed to 1%. We showed in additional analyses that this choice did not affect the conclusions (results not shown). 

The uncertainty of PK parameter estimates was described by the relative standard errors (SE%) from importance sampling. Additionally, a nonparametric bootstrap with 200 replicates was used to quantify the uncertainty. Each bootstrap dataset contained the observations of 18 randomly drawn subjects (i.e., eight patients with CF and ten healthy volunteers) from the original dataset; subjects could be drawn multiple times (i.e., with replacement). We calculated the medians and nonparametric 95% confidence intervals (i.e., the 2.5th to 97.5th percentile) for each PK parameter based on the 200 bootstrap replicates.

*Model comparison and computation*: We compared competing models by their predictive performance assessed via visual predictive checks, the objective function (negative log-likelihood in S-ADAPT), normalized prediction distribution error and other standard diagnostic plots as described previously [11,28,36,37,38]. The importance sampling algorithm (pmethod = 4) in the S-ADAPT software (version 1.57) [39] was used for all population modelling with the SADAPT-TRAN package as facilitator [40,41]. Phoenix/WinNonlin Professional (version 8.1.0, Certara L.P., Princeton, NJ, USA) was used for non-compartmental analysis and statistics.

## 3. Results

Patients with CF were younger, approximately 32% to 40% smaller and leaner than our healthy volunteers based on WT, FFM and lean body mass (LBM, Table 1). Males and females were balanced for our healthy volunteer group; however, the majority (6 of 8) of our patients with CF was female. 

Non-compartmental analysis without body size adjustment showed an approximately 19% larger unscaled renal clearance and an approximately 28% smaller nonrenal clearance in patients with CF compared to those in healthy volunteers (Table 2). The unscaled total clearance was 25% smaller in patients with CF, since renal elimination only accounted for about 6.5%–13% of the total pefloxacin clearance (Table 2). Patients with CF had a comparable volume of distribution, as well as a slightly longer terminal half-life and mean residence time compared to estimates in healthy volunteers.

The area under the plasma concentration time curve was comparable between oral and intravenous dosing within the respective subject group; this indicated a complete extent of bioavailability. The average amount of unchanged pefloxacin recovered in urine was slightly smaller after oral compared to intravenous dosing (Table 2) which could be explained by a small extent of first-pass metabolism of pefloxacin. The fraction excreted as unchanged pefloxacin into urine was approximately 74% larger in patients with CF relative to that in healthy volunteers (Table 2). In contrast, the fractions of dose recovered as norfloxacin and pefloxacin *N*-oxide in urine were similar or only slightly larger in patients with CF compared to those in healthy volunteers.

### Population Pharmacokinetic Modeling

**Figure 2:**
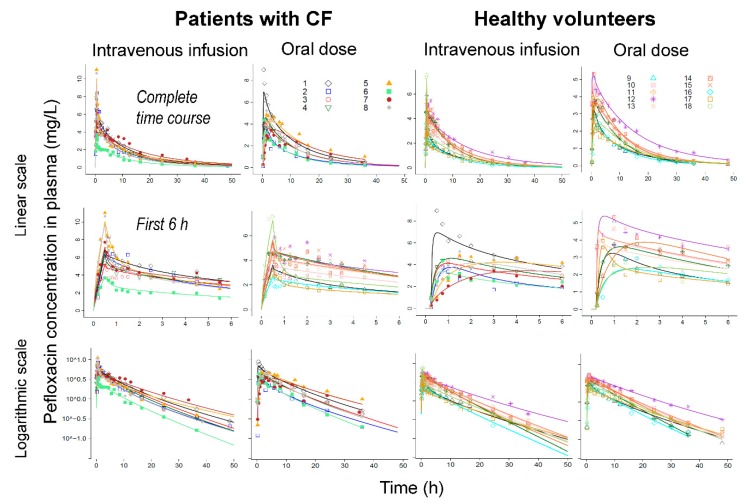
Observed (markers) and individually fitted plasma concentrations (lines) for pefloxacin in patients with CF (left) and healthy volunteers (right) on linear (top and middle rows) and logarithmic (bottom row) scale. The middle row represents the first 6 h on linear scale.

**Figure 3:**
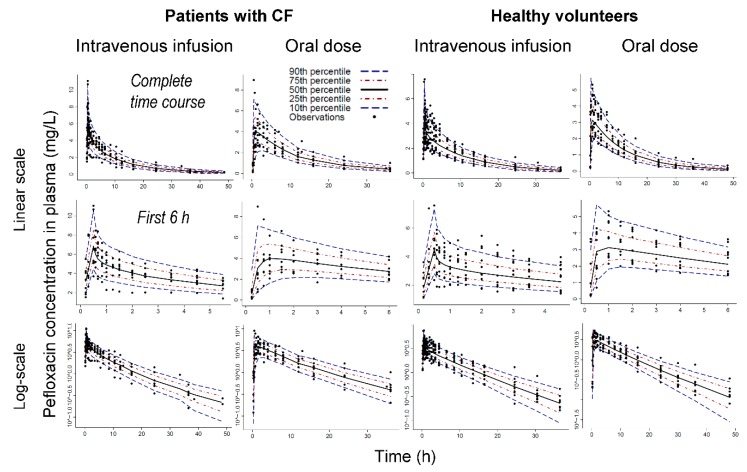
Visual predictive check for pefloxacin plasma concentrations in patients with CF (left) and healthy volunteers (right). The two top rows show plasma concentrations over the complete time course (first row) or during the first 6 h (second row), whereas the third row displays concentrations on logarithmic scale. The plots show the observations (markers), the 50th percentile (i.e., median) of the model predictions (black line) along with the 80% prediction interval [10th to 90th percentile] and the interquartile range [25th to 75th percentile]. Ideally, the median should capture the central tendency of the observations and 10% of the observations should fall outside the 80% prediction interval on either side.

Including a lag-time for oral absorption improved the individual curve fits, predictive performance and the objective function considerably. Models with two or three disposition compartments provided excellent curve fits (Figure 2, Figures S1 and S2) and predictive performance (Figure 3). In the final model (Figure 1), intestinal recirculation represented a distribution process, since all exsorbed pefloxacin was subsequently reabsorbed into the central compartment. When estimated, the fraction of pefloxacin that was reabsorbed became larger than 0.99; thus, this fraction was eventually fixed to 1.0. Including the intestinal recirculation compartment significantly improved the objective function (*p* < 0.0001, likelihood ratio test), especially when the reabsorption half-lives were allowed to differ between patients with CF and healthy volunteers. During initial modeling analyses, CL_GUT_ was estimated to be larger than 100 L/h which exceeds blood flow to gut. Therefore, CL_GUT_ was eventually fixed to this blood flow (66 L/h) and the associated Km_EX_ was estimated. The final model contained three disposition compartments (X1, X2 and X_Int,Cir_; Figure 1). 

*Absorption*: The extent of pefloxacin bioavailability was complete in both subject groups with a BSV of 14.6% CV in patients with CF and 12.7% in healthy volunteers (Table 3). The mean lag-time was the same for both subject groups. However, patients with CF had a slower absorption half-life (19.8 min for patients with CF vs. 11.2 min for healthy volunteers) and the BSV for the absorption half-life was large in both subject groups. Likewise, the reabsorption half-life was longer in patients with CF (Table 3). Estimating different absorption and reabsorption half-lives in both subject groups significantly improved the objective function (*p* = 0.049, likelihood ratio test), improved the curve fits, and was physiologically plausible. 

*Disposition*: Nonrenal clearance accounted for approximately 90% of total clearance (Table 3). While nonrenal clearance was slightly smaller in patients with CF compared to that in healthy volunteers after accounting for body size and body composition, renal clearance was considerably larger in patients with CF. The formation clearance of norfloxacin was comparable between both subject groups, whereas the formation clearance of pefloxacin *N*-oxide was 18% larger in patients with CF (Table 3). 

Exsorption into gut was saturable with a Michaelis-Menten constant of 1.44 mg/L and pefloxacin plasma concentrations exceeded this concentration for over 12 h (Figure 2). For models with linear (i.e., non-saturable) exsorption, the estimated linear exsorption clearance was approximately 3 L/h, but this model had a significantly worse objective function compared to the final model which contained a saturable exsorption (*p* < 0.0001, likelihood ratio test). After accounting for body size and body composition via allometric scaling by FFM, volume of distribution was well comparable between both subject groups. The population mean parameter estimates were reasonably precise (Table 3, Table S1) and the medians from nonparametric bootstrapping matched the estimates from the original dataset (Table S1) closely for most parameters. All 90% confidence intervals included the estimate from the original dataset. 

*Body size models*: Our patients with CF were significantly smaller than our healthy volunteers (Table 1). Therefore, nonrenal and total clearance as well as volume of distribution at steady-state had F_CYF_ estimates considerably below 1.0 when no body size model was applied (Table 4). Both linear and allometric scaling by WT and FFM yielded F_CYF_ close to 1.0 for nonrenal clearance, total clearance and volume of distribution at steady-state. However, renal clearance was estimated to be substantially larger in patients with CF compared to that in healthy volunteers for all body size models (Table 4). Allometric scaling by FFM explained most of the BSV in nonrenal clearance, as well as in the volume of the central and peripheral compartments (Table 5). 

Population PK models that used LBM to describe body size and body composition yielded disease factors slightly more different from 1.0 compared to those that used FFM. Moreover, models based on FFM explained the BSV in nonrenal clearance and volume of distribution of the central compartment slightly better than models based on LBM (results not shown). Given that the population PK model based on allometric scaling by FFM provided excellent curve fits and predictive performance, it was chosen as the final model (Table 3).

## 4. Discussion

This study presents the first PK comparison of the bioavailability and disposition for a primarily nonrenally cleared quinolone antibiotic as well as its two metabolites, norfloxacin and pefloxacin *N*-oxide, between patients with CF and healthy volunteers. With each subject receiving pefloxacin both intravenously and orally in this randomized, crossover study, oral bioavailability could be estimated in each subject. Bioavailability was complete and slightly more variable in patients with CF compared to healthy volunteers (Table 2 and Table 3). Patients with CF have known pathophysiological alterations of the gastrointestinal tract such as bile acid malabsorption, injury of the proximal small intestine and gastric acid hypersecretion [2,44,45,46]. In agreement with results on other quinolones [6,7,9,10,11], we found a longer absorption and reabsorption half-lives of pefloxacin in patients with CF compared to the respective half-lives in healthy volunteers (Table 3). 

Our patients with CF were significantly smaller and leaner than our healthy volunteers (Table 1). After accounting for the differences in body size and body composition via allometric scaling by FFM, nonrenal clearance and volume of distribution were well comparable between both subject groups (Table 4). However, renal clearance of pefloxacin was approximately 53% larger in patients with CF compared to that in healthy volunteers. For ciprofloxacin, most studies report a similar renal clearance in patients with CF compared to that in healthy volunteers (ratios ranging from 0.83 to 1.23 for renal clearance) [4,5,6]. We recently reported a comparable renal clearance for fleroxacin (ratio: 1.12) in both subject groups, but higher renal clearances for its two metabolites in patients with CF (i.e., ratios of 1.53 for *N*-oxide fleroxacin and 1.70 for *N*-demethyl-fleroxacin) [11]. With ciprofloxacin and fleroxacin being primarily renally cleared quinolones, these results may not be directly transferable to pefloxacin which is predominantly non-renally cleared.

Yahiaoui et al. reported the disease spectrum and the clinical relevance of renal involvement in cystic fibrosis [47]. The cystic fibrosis transmembrane conductance regulator (CFTR) is considerably expressed in different parts of the nephron. Inactivation of CFTR can lead to variable but significant low molecular weight proteinuria in patients with CF [48]. Asymptomatic nephrocalcinosis has been reported in up to 90% and hypercalciuria in up to 30% of patients with CF [49]. Fluoroquinolones are known to chelate calcium [14] and a higher calcium concentration in urine might have increased the renal clearance of pefloxacin; however, this potential mechanism needs further investigation.

Given that nonrenal clearance accounted for approximately 90% of total clearance in patients with CF and healthy volunteers, the total clearance was comparable between both subject groups. The BSV of nonrenal and total clearance as well as of volume of distribution was best explained by allometric scaling via FFM (Table 5) suggesting that FFM was a useful descriptor of body size and body composition for pefloxacin as a nonrenally cleared fluoroquinolone.

The estimates of our PK parameters for healthy volunteers (Table 2 and Table 3) were in agreement with those from prior studies [50,51,52,53]. We are, however, not aware of any study on the PK of pefloxacin in patients with CF. For other quinolones, most prior studies reported similar renal and nonrenal clearances in patients with CF and healthy volunteers [4,6,9,10,11,12]. We estimated the metabolite formation clearance (Figure 1) based on the amounts of metabolite excreted into urine. The oral bioavailability of norfloxacin (i.e., one of the metabolites) is approximately 30 to 40% and renal clearance of norfloxacin presents its primary route of elimination [13,14]. The second metabolite, pefloxacin *N*-oxide, is also likely primarily renally eliminated, given the extensive renal clearance reported for *N*-oxide fleroxacin [11]. If these two metabolites had a small additional nonrenal clearance, the metabolite formation clearance would be slightly larger than that reported in Table 3. A reversible metabolism has been previously reported for the *N*-oxide metabolite of some quinolones [17,54]; however, the extent of reversibility was small and our available data did not allow us to estimate a potential reversible metabolism. For fleroxacin, the formation clearances of *N*-oxide fleroxacin and for *N*-demethyfleroxacin were both approximately 38% larger in patients with CF compared to those in healthy volunteers [11]. In contrast, the formation clearance was only 18% larger for pefloxacin *N*-oxide in patients with CF and similar between both subject groups for norfloxacin (Table 3). Future studies on other quinolones are necessary to further investigate the extent of metabolite formation in patients with CF. 

Exsorption of quinolones into intestine has been identified via charcoal studies in animals and man [11,13,14,33,34,55]. Some quinolones show extensive elimination into feces (i.e., 10%–15% of the fleroxacin dose and 60% of sparfloxacin dose) [33,34] which can however not be explained by the rather small fractions of dose (1%–3%) recovered in bile [13,14]. These data clearly suggest the presence of exsorption of quinolones into intestine. The latter was implemented in a prior population PK analysis for fleroxacin [11] via a saturable exsorption clearance, since the extent of biliary excretion was too small to explain the amount of drug recovered in feces. Pefloxacin displayed a weak inhibition of human organic cation transporter 1 (hOCT1) [56] and one study showed that pefloxacin and ciprofloxacin show a transporter-related interaction affecting the intestinal clearance [57]. Other quinolones are secreted by a variety of transporters, including MDR1 and MRP2 for grepafloxacin [58,59,60], MDR1 for sparfloxacin [61], as well as OCT1 and BCRP for ciprofloxacin [57,62,63].

Given the complete bioavailability of pefloxacin, our model assumed that exsorbed pefloxacin is completely reabsorbed into the central compartment (Figure 1). Therefore, the intestinal recirculation compartment represented a distribution site. The isoelectric points of pefloxacin (pI: 6.95) and of fleroxacin (6.78) are considerably lower than those of ciprofloxacin (pI: 7.42) and sparfloxacin (pI: 7.53). This leads to a higher fraction of zwitterionic and uncharged molecules in the slightly acidic pH in small intestine for pefloxacin and fleroxacin [14,57]. Likewise, pefloxacin is more lipophilic than ciprofloxacin and fleroxacin which further contributes to the complete bioavailability of pefloxacin. A major part of pefloxacin is recovered in feces [16] and pefloxacin reaches high concentrations in this matrix [64,65]. While these data show that pefloxacin is present at considerable concentrations in the gut and ultimately in feces, they also suggest that reabsorption of pefloxacin is less than 100%. 

Our study design did not allow us to estimate the reabsorption fraction in the absence of an additional treatment arm with activated charcoal blocking reabsorption. While this presents a potential limitation for the exsorption/reabsorption part of our model, the effect of activated charcoal on the nonrenal clearance of other quinolones has been shown previously in healthy volunteers [13,14,16]. Other limitations of our study include the moderately small sample size and the lack of plasma concentration data of the two metabolites. The latter did not allow us to estimate the elimination clearances and volumes of distribution of the metabolites. We estimated the fraction of drug in urine based on the cumulative amount excreted until the last collection interval, but did not have the individual amounts excreted during each urine collection interval available. While this presents a limitation of the present analysis, this study could provide valuable insights on the comparison of bioavailability and disposition of pefloxacin between patients with CF and healthy volunteers while leveraging a population PK modeling approach.

## 5. Conclusions

This study was the first to compare the PK of a primarily nonrenally cleared quinolone between patients with CF and healthy volunteers. We studied pefloxacin in a randomized, two-way crossover study with intravenous and oral dosing. While bioavailability was complete in both subject groups, the absorption and reabsorption half-lives were longer in patients with CF compared to those in healthy volunteers. Differences in body size and body composition were best described by allometric scaling based on FFM. This body size model also better explained the random BSV compared to the other tested body size models. The body size adjusted nonrenal and total clearance as well as volume of distribution at steady state were well comparable between both subject groups. However, renal clearance of pefloxacin was considerably and significantly larger in patients with CF. The metabolite formation clearance was well comparable for norfloxacin and 18% larger in patients with CF for pefloxacin *N*-oxide compared to estimates in healthy volunteers. Overall, this study showed that the PK of pefloxacin as a predominantly nonrenally cleared quinolone was comparable after accounting for differences in body size and body composition between patients with CF and healthy volunteers.

## Figures and Tables

**Table 1:** Demographic data (median [range]) of patients with CF and healthy volunteers.

Demographic Variable	Patients with CF	Healthy Volunteers
Number of subjects (males/females)	8 (2/6) ^c^	10 (5/5)
Age (year)	19 [17–24] ^d^	24 [18–27]
Height (cm)	166 [158–175] ^d^	174 [168–191]
Total body weight (WT) (kg)	46.3 [35.5–63.5] ^d^	77.5 [55.0–82.0]
Fat-free mass (FFM) ^a^ (kg)	33.3 [27.3–46.4] ^d^	52.1 [37.7–64.0]
Lean body mass (LBM) ^b^ (kg)	38.0 [31.0–47.1] ^d^	55.6 [43.0–64.5]
Body mass index (kg m^−2^)	17.6 [13.4–22.2] ^d^	21.6 [19.5–27.3]

^a^ Calculated based on the formula by Janmahasatian et al. [26]. ^b^ Calculated based on the formula by Cheymol and James [42,43]. ^c^ *p* = 0.37, Fisher’s exact test, two-tailed. ^d^ *p* < 0.01, unpaired *t*-test (two-tailed) with unequal variance.

**Table 2:** Pharmacokinetic parameters for oral and intravenous pefloxacin in patients with CF and healthy volunteers from non-compartmental analysis (data shown as median [range]).

Pharmacokinetic Parameter	Patients with CF		Healthy Volunteers	
	Oral	Intravenous	Oral	Intravenous
Total clearance (L/h)	6.47 [3.50–10.4]	6.47 [3.81–12.6]	8.81 [4.76–12.6]	8.35 [5.56–16.0]
Renal clearance (L/h)	0.650 [0.442–0.890]	0.796 [0.457–1.08]	0.564 [0.426–0.870]	0.654 [0.395–1.01]
Nonrenal clearance (L/h)	5.65 [3.06–9.97]	5.74 [3.23–11.8]	8.32 [4.24–12.0]	7.60 [5.17–15.0]
Volume of distribution at steady-state (L)	105 [73.0–142]	98.1 [82.2–166]	105 [91.1–200]	98.2 [80.5–202]
Time to peak concentration (h)	1.13 [0.50–3.00]	0.50 [0.50–0.67]	1.50 [0.50–4.00]	0.54 [0.50–1.50]
Peak concentration (mg/L)	4.49 [3.45–8.94]	7.40 [3.68–11.0]	4.19 [2.68–5.32]	4.76 [2.35–7.56]
Terminal half-life (h)	11.7 [9.58–19.2]	12.3 [10.6–20.1]	11.7 [7.95–15.9]	9.40 [6.18–12.4]
Mean residence time (h)	15.4 [13.3–25.5]	15.4 [13.0–25.1]	14.1 [10.4–20.1]	13.2 [9.85–16.2]
Oral bioavailability (%)	107 [58–178]		99 [80–141]	
Area under the plasma concentration time curve (mg·h/L)	61.8 [38.4–114]	61.8 [31.7–105]	45.4 [31.6–84.0]	48.0 [25.0–71.9]
Fraction of dose excreted as pefloxacin in urine (%)	11.1 [4.24–15.6]	13.2 [6.83–17.5]	6.54 [4.73–10.9]	7.44 [6.30–10.2]
Fraction of dose excreted as norfloxacin in urine (%)	15.9 [9.10–20.0]	14.8 [8.77–27.1]	15.3 [10.7–17.7]	14.0 [11.6–22.4]
Fraction of dose excreted as pefloxacin *N*-oxide in urine (%)	18.3 [16.3–20.5]	18.6 [15.4–21.7]	17.2 [11.8–18.3]	14.8 [10.3–17.2]

**Table 3:** Population PK parameter estimates for pefloxacin, norfloxacin and pefloxacin *N*-oxide in patients with CF and healthy volunteers for a model with allometric scaling by FFM.

PK Parameters	Symbol	Unit	Patients with CF		Healthy Volunteers	
			Pop. Mean (SE%)	BSV ^a^ (SE%)	Pop. Mean (SE%)	BSV ^a^ (SE%)
Pefloxacin						
Oral bioavailability	F_BIO_	-	1.00 (5.60%)	0.146 (109%)	1.03 (4.70%)	0.127 (48.2%)
Absorption lag-time	T_lag_	min	13.3 (4.00%)	0.0712 (121%)	13.3 (4.00%)	0.0712 (121%)
Absorption half-life	T_abs_	min	19.8 (36.9%)	1.10 (54.1%)	11.2 (31.0%)	0.983 (114%)
Reabsorption half-life from intestine	T_reabs_	min	65.4 (15.3%)	0.334 (83.1%)	20.8 (19.3%)	0.804 (107%)
Volume of distribution for central compartment	V1 ^b^	L	37.4 (19.6%)	0.435 (40.9%)	40.8 (12.9%)	0.435 (40.9%)
Volume of distribution for peripheral compartment	V2 ^b^	L	59.9 (15.5%)	0.131 (194%)	65.4 (5%)	0.131 (194%)
Total clearance	CL_TOT_^b^	L/h	8.44 (12.8%) ^c^	-	9.26 (7.10%) ^c^	-
Non-renal clearance	CL_NR_^b^	L/h	7.37 (14.5%)	0.238 (35.0%)	8.56 (7.70%)	0.238 (35.0%)
Renal clearance	CL_R_^b^	L/h	1.07 (14.1%)	0.168 (44.6%)	0.705 (6.50%)	0.168 (44.6%)
Distribution clearance	CL_D_^b^	L/h	406 (33.7%)	1.19 (51.8%)	406 (33.7%)	1.19 (51.8%)
Gut clearance for enterohepatic circulation	CL_GUT_^b^	L/h	66 (fixed)	0 (fixed)	66 (fixed)	0 (fixed)
Plasma concentration associated with half-maximal CL_GUT_	Km_EX_	mg/L	1.44 (12.0%)	0.1 (fixed)	1.44 (12.0%)	0.1 (fixed)
**Norfloxacin**						
Formation fraction	fm_NOR_	-	0.201 ^d^	[0.152 to 0.313] ^d^	0.174 ^d^	[0.147 to 0.195] ^d^
Formation clearance	CLf_NOR_^b^	L/h	1.48 ^e^		1.49 ^e^	
**Pefloxacin *N*-oxide**						
Formation fraction	fm_NOX_	-	0.244 ^d^	[0.171 to 0.272] ^d^	0.178 ^d^	[0.151 to 0.205] ^d^
Formation clearance	CLf_NOX_^b^	L/h	1.80 ^e^		1.52 ^e^	

The additive and proportional residual errors of plasma concentrations were 0.00984 mg/L and 15.1% for pefloxacin. The additive residual error of the fraction excreted in urine were fixed to 1% for pefloxacin, norfloxacin and pefloxacin *N*-oxide. ^a^ Between subject variability (BSV); estimates represent apparent coefficients of variation of a normal distribution on natural logarithmic scale. The numbers in parentheses are the relative standard errors (SE%) of the estimated variance. ^b^ All volume and clearance parameter estimates represent group estimates for subjects with a standard body size (i.e., FFM of 53 kg). Volumes of distribution were scaled with an exponent of 1.0 (fixed) and clearances used a fixed exponent of 0.75. ^c^ Calculated as the sum of CL_NR_ and CL_R_. Standard errors were calculated via error propagation. ^d^ For logistically transformed parameters (i.e., fm_NOR_, fm_NOX_), the median and range of individual subject estimates are provided. ^e^ Calculated as the product of the mean linear nonrenal clearance of pefloxacin (CL_NR_) and the formation fraction for norfloxacin (fm_NOR_) or that of pefloxacin N-oxide (fm_NOX_; Figure 1). The formation clearances are not directly estimated model parameters.

**Table 4:** Disease specific scale factors which represent the clearance (or volume of distribution) in patients with CF divided by that in healthy volunteers after accounting for body size and body composition by the respective size model.

Body Size Model ^a^	F_CYF, CLNR_	F_CYF, CLR_	F_CYF, CLT_	F_CYF, VSS_
No body size model	0.649 (34.8%)	1.17 (28.5%)	0.688 ^b^	0.568 (22.6%)
WT linear scaling	0.983 (9.5%)	1.75 (10.0%)	1.04	0.948 (10.2%)
WT allometric	0.891 (6.7%)	1.59 (8.2%)	0.944	0.926 (6.9%)
FFM linear scaling	0.956 (7.1%)	1.67 (8.3%)	1.01	0.976 (6.6%)
FFM allometric	0.861 (12.3%)	1.53 (12.5%) ^c^	0.912	0.916 (14.7%)

^a^ This table compares the different body size models for subjects of standard body size (i.e., a WT_STD_ of 70 kg or FFM_STD_ of 53 kg). An ideal body size model should explain the differences in body size and body composition and thus yield disease specific scale factors close to 1.0. ^b^ The ratio of total clearance between patients with CF and healthy volunteers (F_CYF, CLT_) was calculated as weighted average of F_CYF, CLNR_ and F_CYF, CLR_ (with nonrenal clearance being the predominant clearance of pefloxacin). The F_CYF, CLT_ was not an estimated model parameter. ^c^ Statistically significantly higher than 1.0 (*p* < 0.01, from 200 nonparametric bootstrap replicates). While no bootstrap was performed for the other body size models, it is highly likely that renal clearance would have also been significantly higher in patients with CF for linear and allometric scaling by WT and linear scaling by FFM.

**Table 5:** Comparison of between-subject variability estimates between different body size models.

Body Size Model	Relative between Subject Variance (%)				
	CL_NR_	CL_R_	CL_TOT_	V1	V2
WT linear scaling	100 ^a^	100	100	100	100
WT allometric	108 ^b^	87	107	103	94
FFM linear scaling	64 ^b,c^	207 ^d^	67 ^d^	60	108
FFM allometric	79 ^b,c^	155 ^d^	80 ^d^	74	67

This table reports the variance of the respective body size model divided by the variance of linear scaling by total body weight (see Table 3 for parameter explanations). ^a^ The between-subject variances were reported relative to the variance for linear scaling by WT. ^b^ A lower relative variance indicates that the unexplained (i.e., random) variability was reduced by the tested body size and body composition model. ^c^ These values mean that the between subject variance was reduced by 36% for linear scaling and by 21% for allometric scaling based on FFM, both compared to linear scaling by WT. ^d^ Renal clearance was much smaller than nonrenal clearance and the estimated BSV of renal clearance was also smaller than the BSV of nonrenal clearance. Therefore, the high relative variances for renal clearance had minimal impact on the relative variances for total clearance.

## Supplementary Materials

The following are available online at https://www.mdpi.com/1999-4923/11/7/323/s1, Figure S1: Observed vs. individual fitted plasma concentrations (top) and fractions of dose excreted in urine (rows 2 to 4) on linear (left side) and logarithmic scale (right side). The green line represents the line of identity and the dashed blue line a LOESS smoother, Figure S2: Observed vs. population fitted plasma concentrations (top) and fractions of dose excreted in urine (rows 2 to 4) on linear (left side) and logarithmic scale (right side). The green line represents the line of identity and the dashed blue line a LOESS smoother, Figure S3: Estimation code for the final model in SADAPT-TRAN format, Table S1: Estimates from the original dataset compared to the median and 95% confidence intervals from nonparametric bootstrapping (200 replicates) for the final population PK model.
