# Supplementary material for: Comparable Bioavailability and Disposition of Pefloxacin in Patients with Cystic Fibrosis and Healthy Volunteers Assessed via Population Pharmacokinetics
Source: Pharmaceutics. 2019 Jul 10;11(7):323. doi: 10.3390/pharmaceutics11070323 (PMC6681055; doi:10.3390/pharmaceutics11070323)
Supplement: Supplementary file 1 [file pharmaceutics-11-00323-s001.pdf]

Supplementary Materials

Comparable Bioavailability and Disposition of Pefloxacin in Patients with Cystic Fibrosis and Healthy Volunteers Assessed via Population Pharmacokinetics

Jürgen B. Bulitta, Yuanyuan Jiao, Cornelia B. Landersdorfer, Dhruvitkumar S. Sutaria, Xun Tao, Eunjeong Shin, Rainer Höhl, Ulrike Holzgrabe, Ulrich Stephan and Fritz Sörgel

Table 1. Estimates from the original dataset compared to the median and 95% confidence intervals from nonparametric bootstrapping (200 replicates) for the final population PK model.

PK Parameters	Symbol	Unit	Population Mean		Between Subjects Variability (CV)	
			Original Dataset	Median (95% CI) (2.5th to 97.5th percentile)	Original Dataset	Median (95% CI) (2.5th to 97.5th percentile)
Pefloxacin						
Oral bioavailability	F_{BIO}	-	1.00 [a]<br>1.03 [b]	1.01 (0.909–1.11) [a]<br>1.03 (0.934–1.10) [b]	0.146 [a]<br>0.127 [b]	0.140 (0.0548–0.210) [a]<br>0.119 (0.0478–0.160) [b]
Absorption lag-time	T_{lag}	min	13.3	13.4 (12.8–14.1)	0.0712	0.0631 (0.0261–0.0867)
Absorption half-life	T_{abs}	min	19.8 [a]<br>11.2 [b]	20.2 (9.65–45.1) [a]<br>11.3 (6.20–18.1) [b]	1.10 [a]<br>0.983 [b]	1.01 (0.627–1.35) [a]<br>0.969 (0.518–1.34) [b]
Reabsorption half-life from intestine	T_{reabs}	min	65.4 [a]<br>20.8 [b]	65.5 (41.0–89.7) [a]<br>20.0 (6.37–41.8) [b]	0.334 [a]<br>0.804 [b]	0.0720 (0.00768–0.600) [a]<br>0.747 (0.0965–1.27) [b]
Volume of distribution for central comp.	V1	L	40.8	39.8 (22.2–58.6)	0.435	0.417 (0.0313–0.632)
Volume of distribution for peripheral comp.	V2	L	65.4	66.6 (50.2–76.7)	0.131	0.0619 (0.00851–0.309)
Non-renal clearance	CL_{NR}	L/h	8.56	8.59 (7.44–9.77)	0.238	0.224 (0.125–0.310)
Renal clearance	CL_{R}	L/h	0.705	0.710 (0.662–0.753)	0.168	0.166 (0.0111–0.265)
Distribution clearance	CL_{D}	L/h	406	414 (164–1,199)	1.19	1.18 (0.693–2.05)
Disease factor for nonrenal clearance	$F_{CYF, NR}$	-	0.861	0.850 (0.694–1.07)	0.05	fixed
Disease factor for renal clearance	$F_{CYF, R}$	-	1.53	1.53 (1.21–1.78) [c]	0.05	fixed
Disease factor for volume of distribution	$F_{CYF, VSS}$	-	0.916	0.931 (0.739–1.15)	0.05	fixed
Maximum rate of exsorption	CL_{gut}	L/h	66.0	66.0 (fixed)	0	fixed
Plasma conc. associated with half-maximal Vm_{EX}	Km_{EX}	mg/L	1.44	1.50 (0.711–2.50)	0.1	fixed
Norfloxacin						
Formation fraction	fm_{NOR}	-	0.201 [a]<br>0.174 [b]	0.198 (0.168–0.239) [a]<br>0.172 (0.162–0.185) [b]	-	-
Pefloxacin-N-oxide						
Formation fraction	fm_{NOX}	-	0.244 [a]<br>0.178 [b]	0.247 (0.220–0.271) [a]<br>0.180 (0.167–0.190) [b]	-	-

[a] Parameter estimates for patients with CF.

[b] Parameter estimates for healthy volunteers. When only one line of parameter estimates is reported, the same estimate was used in patients with CF and healthy volunteers.

[c] Significantly different from 1.0 ($p < 0.05$; two-sided test) based on 200 bootstrap replicates.

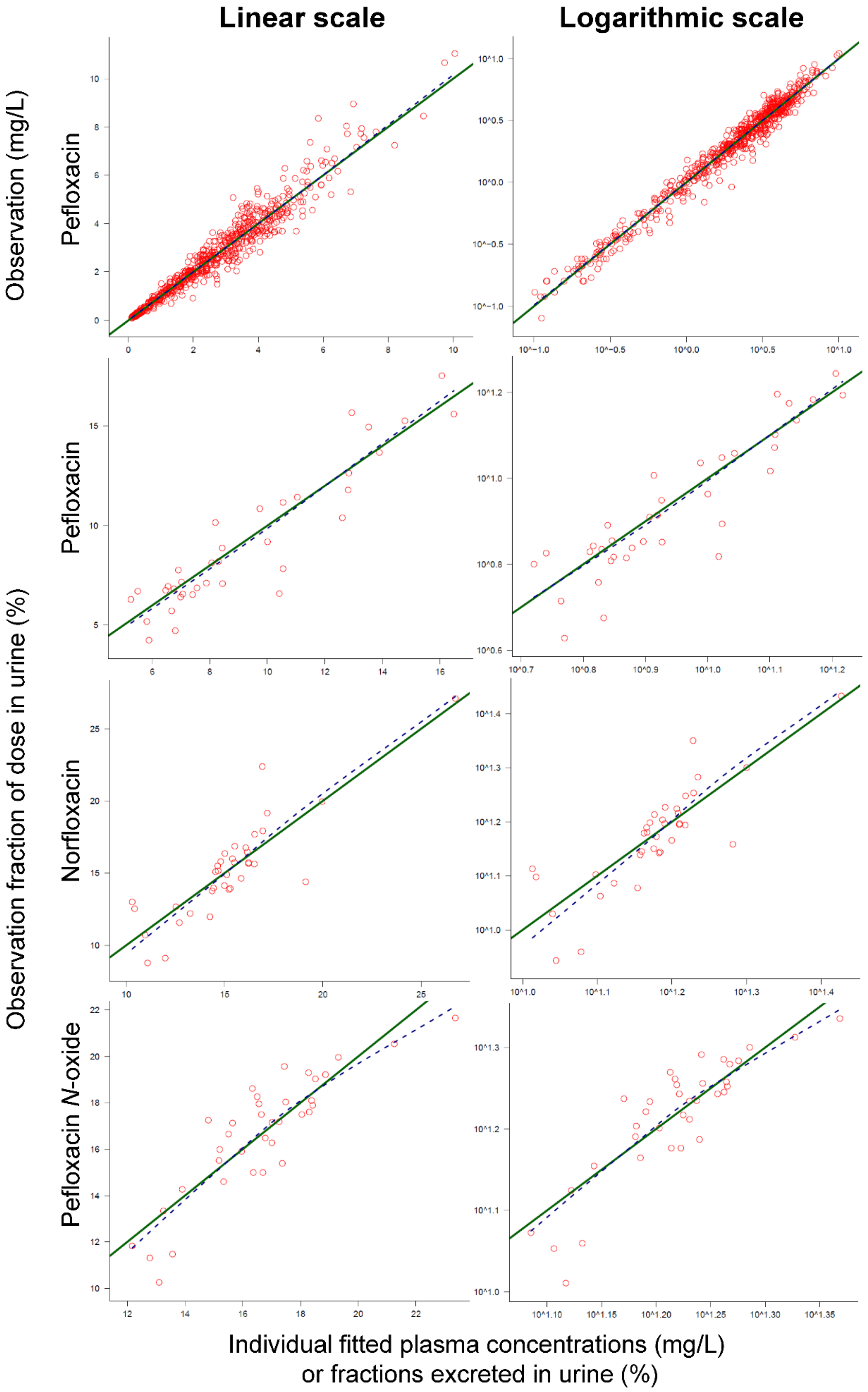

Figure S1. Observed vs. individual fitted plasma concentrations (top) and fractions of dose excreted in urine (rows 2 to 4) on linear (left side) and logarithmic scale (right side). The green line represents the line of identity and the dashed blue line a LOESS smoother.

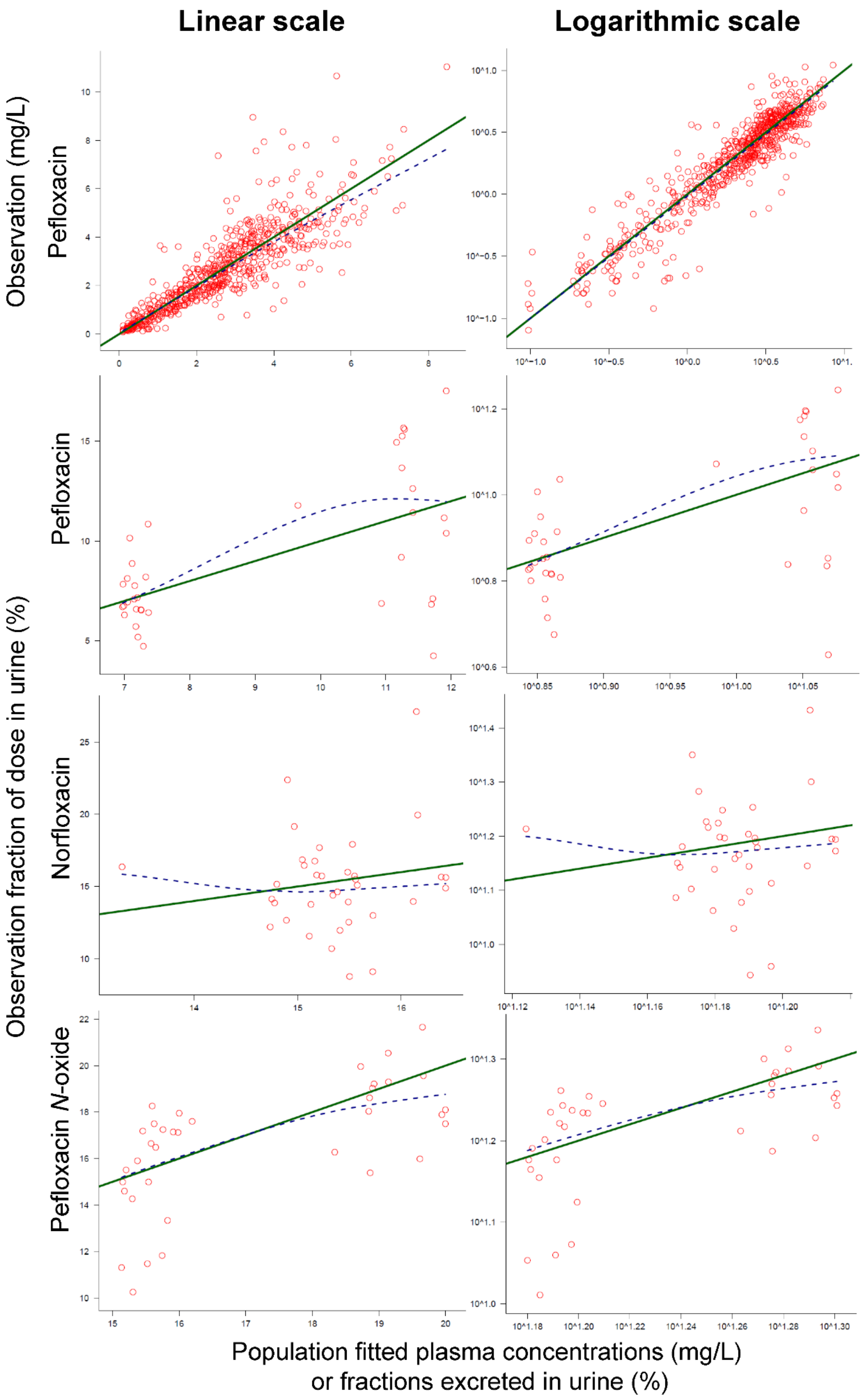

Figure S2. Observed vs. population fitted plasma concentrations (top) and fractions of dose excreted in urine (rows 2 to 4) on linear (left side) and logarithmic scale (right side). The green line represents the line of identity and the dashed blue line a LOESS smoother.

```
$PROJECT THIS WILL BE A TERRIFIC PROJECT

$DIFFEQ_DIF

GUT    = X(1)
DC1    = X(2)/V1_COV
DC2    = X(3)/V2_COV
URI    = X(4) ; Amount of pefloxacin         in urine
URINO  = X(5) ; Amount of norfloxacin        in urine
URIDE  = X(6) ; Amount of pefloxacin-N-oxide in urine
GUTEHC = X(7)

TPD = MOD(T,72)

IF (TPD.GT.TLAGH) THEN
   KAT = KA
ELSE
   KAT = 0
ENDIF

CLMM = CLgut_COV*KM / (KM+DC1)

XP(1)  =       - KAT*GUT
XP(2)  =  R(1) + KAT*GUT - CLR_COV*DC1 - CLNR_COV*DC1 - CLMM*DC1 - CLD_COV*DC1 + CLD_COV*DC2 + KAEHC*GUTEHC
XP(3)  =                                                           CLD_COV*DC1 - CLD_COV*DC2

XP(4)  =                   CLR_COV*DC1
XP(5)  =                          FM_NOR*CLNR_COV*DC1
XP(6)  =                          FM_NOX*CLNR_COV*DC1

XP(7)  =                                              + CLMM*DC1                             - KAEHC*GUTEHC

$OUTPUT_GLB

IF (GRP.EQ.1) THEN
 ; Patients with Cystic Fibrosis
   FBIO       = FBIO_CF
   KA         = LOG(2)/(TABS_CF/60)
   KAEHC      = LOG(2)/(TAEHC_CF/60)

   FCYF_CLR   = FCLR
   FCYF_CLNR  = FCLNR
   FCYF_CLEX  = FCLEX
   FCYF_VSS   = FVSS

   TFM_NOR    = TFM_NORCF
   DFM_NOX    = DFM_NOXCF
ELSE
 ; Healthy volunteers
   FBIO       = FBIO_HV
   KA         = LOG(2)/(TABS_HV/60)
   KAEHC      = LOG(2)/(TAEHC_HV/60)

   FCYF_CLR   = 1
   FCYF_CLNR  = 1
   FCYF_CLEX  = 1
   FCYF_VSS   = 1

   TFM_NOR    = TFM_NORHV
   DFM_NOX    = DFM_NOXHV
ENDIF

```

Figure S3. code for the final model in SADAPT-TRAN format.

```
BOLUSF(1) = FBIO

FM_NOR      = 1/(1+EXP(-TFM_NOR))

TFM_NORNOX = DFM_NOX + TFM_NOR
FM_NORNOX  = 1/(1+EXP(-TFM_NORNOX))

FM_NOX2     = FM_NORNOX - FM_NOR
FM_NOX      = MAX(0,FM_NOX2) ; to improve robustness of code

TLAGH= TLAG/60

FWTCL  = (FFM/53)**0.75
FWTV   = (FFM/53)

; Elimination clearances
CLR_COV      = FCYF_CLR  * FWTCL * CLR
CLNR_COV     = FCYF_CLNR * FWTCL * CLNR
CLgut_COV    = FCYF_CLEX * FWTCL * CLgut
CLD_COV      =             FWTCL * CLD
V1_COV       = FCYF_VSS  * FWTV  * V1
V2_COV       = FCYF_VSS  * FWTV  * V2

$OUTPUT_ICS

$OUTPUT_EQN

IF (X(1).LT.0) X(1) = 0
IF (X(2).LT.0) X(2) = 0
IF (X(3).LT.0) X(3) = 0
IF (X(4).LT.0) X(4) = 0
IF (X(5).LT.0) X(5) = 0
IF (X(6).LT.0) X(6) = 0
IF (X(7).LT.0) X(7) = 0

C1   = X(2)/V1_COV ; Serum concentrations of pefloxacin (mg/L)

Y(1) = C1

Y(2) = 100*X(4)/400  ; fraction of dose excreted in urine as unchanged pefloxacin
Y(3) = 100*X(5)/400  ; fraction of dose excreted in urine as norfloxacin
Y(4) = 100*X(6)/400  ; fraction of dose excreted in urine as pefloxacin-N-oxide

; Empty the bucket for the cumulative amount excreted into urine (=reset event)
IF (EVID.EQ.5) THEN
   X(1) = 0
   X(2) = 0
   X(3) = 0
   X(4) = 0
   X(5) = 0
   X(6) = 0
   X(7) = 0
ENDIF

$VARMOD_EQN

V(1) = ( SDin + SDsl*Y(1) ) * ( SDin + SDsl*Y(1) )

V(2) = UPin * UPin
V(3) = URin * URin
V(4) = UXin * UXin

$POPMOD_EQN

```

Figure S3. code for the final model in SADAPT-TRAN format (**continued**).
